# Pentagalloyl Glucose (PGG) Exhibits Anti-Cancer Activity against Aggressive Prostate Cancer by Modulating the ROR1 Mediated AKT-GSK3β Pathway

**DOI:** 10.3390/ijms25137003

**Published:** 2024-06-26

**Authors:** Vignesh Sivaganesh, Tram M. Ta, Bela Peethambaran

**Affiliations:** 1 Department of Biology, Saint Joseph’s University, 600 S 43rd St, Philadelphia, PA 19104, USA; vs271231@pcom.edu (V.S.); tt20802863@sju.edu (T.M.T.); 2 Department of Biomedical Sciences, Philadelphia College of Osteopathic Medicine, 4170 City Ave, Philadelphia, PA 19131, USA

**Keywords:** ROR1, receptor tyrosine kinase, pentagalloyl glucose, targeted therapy, prostate cancer, castration resistance, oncology, natural small molecule inhibitor

## Abstract

Androgen-receptor-negative, androgen-independent (AR^neg^-AI) prostate cancer aggressively proliferates and metastasizes, which makes treatment difficult. Hence, it is necessary to continue exploring cancer-associated markers, such as oncofetal Receptor Tyrosine Kinase like Orphan Receptor 1 (ROR1), which may serve as a form of targeted prostate cancer therapy. In this study, we identify that Penta-O-galloyl-β-D-glucose (PGG), a plant-derived gallotannin small molecule inhibitor, modulates ROR1-mediated oncogenic signaling and mitigates prostate cancer phenotypes. Results indicate that ROR1 protein levels were elevated in the highly aggressive AR^neg^-AI PC3 cancer cell line. PGG was selectively cytotoxic to PC3 cells and induced apoptosis of PC3 (IC_50_ of 31.64 µM) in comparison to normal prostate epithelial RWPE-1 cells (IC_50_ of 74.55 µM). PGG was found to suppress ROR1 and downstream oncogenic pathways in PC3 cells. These molecular phenomena were corroborated by reduced migration, invasion, and cell cycle progression of PC3 cells. PGG minimally and moderately affected RWPE-1 and AR^neg^-AI DU145, respectively, which may be due to these cells having lower levels of ROR1 expression in comparison to PC3 cells. Additionally, PGG acted synergistically with the standard chemotherapeutic agent docetaxel to lower the IC_50_ of both compounds about five-fold (combination index = 0.402) in PC3 cells. These results suggest that ROR1 is a key oncogenic driver and a promising target in aggressive prostate cancers that lack a targetable androgen receptor. Furthermore, PGG may be a selective and potent anti-cancer agent capable of treating ROR1-expressing prostate cancers.

## 1. Introduction

Prostate cancer has a complex molecular pathway that regulates its progression. Initially, prostate cancer relies on the androgen receptor (AR) to elicit survival and metastasis [1,2]. Hence, clinicians seek to target AR pathways through medications that inhibit the production of testosterone in the testes (androgen deprivation therapy) [1,2,3]. In androgen deprivation therapy (a form of hormone therapy), testosterone and dihydrotestosterone binding to AR is reduced, thus the androgen receptor cannot translocate to the nucleus, bind to androgen response elements (ARE) in nuclear DNA, and subsequently elicit transcription of growth-promoting genes [1,2]. However, the use of androgen deprivation therapy often results in the formation of castration-resistant prostate cancer (CRPC) [3,4]. CRPC operates through many modalities including AR amplification, constitutively active splice variants, sensitization via post-translational modifications, receptor mutations, and enhanced co-stimulator activity, which allow CRPC to survive despite reduced androgen production or ligand-based activation of AR [3,5]. These types of CRPCs can be molecularly classified as AR-positive, androgen-independent cancers (AR^pos^-AI), since they can proliferate via mechanisms that do not rely on ligand-based AR activation, but still rely on AR signaling overall [3,5]. Hormone therapies that suppress systemic androgen production (e.g., abiraterone) or block testosterone from binding to the androgen receptor (e.g., enzalutamide) are used to treat CRPC and have caused an increase in the percentage of metastatic CRPC patients who have AR-negative cancer (from 11% to 36%) [4,6,7]. These prostate tumors, which can be classified as AR-negative, androgen-independent (AR^neg^-AI) cancer, are a subtype of CRPC that have more aggressive characteristics than AR^pos^-AI prostate cancer and are modeled by established cell lines PC3 and DU145 [8,9].

Currently, targeted therapies used for the treatment of metastatic CRPC include prostatic acid phosphatase (PAP) immunotherapy, known as sipuleucel-T; prostate-specific membrane antigen (PSMA)-targeting radioligand therapy, known as Lutetium-177-PSMA-617; programmed cell death protein 1 (PD-1) inhibitor immunotherapy, known as pembrolizumab; and polyadenosine-diphosphate-ribose polymerase (PARP) inhibitors (oliparib, rucaparib) [10,11,12]. However, there are limitations to these forms of therapies. For example, evidence suggests that PAP is also highly expressed in normal prostate tissue and can also be expressed in other cell types, which warrants further investigation into whether sipuleucel-T can cause off-target toxicity [13,14,15]. PSMA exhibits marked intratumor and interpatient heterogeneity, which means that patients with high PSMA variability within a tumor and across different metastatic nodules may be resistant to this form of therapy [16,17]. PD-1 immune checkpoint inhibition seems to be effective mainly in patients who harbor metastatic CRPC with microsatellite instability (MSI) or mismatch repair (MMR) gene deficiency [11,12,18]. However, only around 1% of patients have germline MMR deficiency, while about 5% have somatic MMR mutations or MSI-high status, which means only a small subset of patients may benefit the most from this form of therapy [18,19,20,21,22,23]. PARP inhibitors are more effective when the prostate tumor exhibits a mutation in DNA damage repair genes such as breast cancer gene 2 (BRCA2), BRCA1, and ataxia-telangiectasia mutated serine/threonine kinase (ATM) [10,11,24]. More specifically, the greatest benefit is seen in patients who exhibit a biallelic BRCA2 knockout mutation [10,24]. Given that only 8–16% of metastatic CRPC harbor germline mutations and 27% of metastatic CRPC have both germline or somatic mutations of DNA repair genes, PARP inhibitor therapy may be effective in a small subset of patients [19,24,25,26,27]. Despite the potential limitations, these therapies are used for metastatic CRPC, especially when other treatment modalities have been exhausted. One of the primary treatments for metastatic prostate cancer is chemotherapy (e.g., docetaxel, cabazitaxel), which can be provided in combination with hormone therapy [28]. However, chemotherapeutic agents can be highly toxic to normal cells, which often leads to adverse effects in patients and discontinuation of therapy [29,30,31]. Hence, it is crucial to find more molecular targets that can be used to treat aggressive prostate cancer while minimally harming normal tissue. Additionally, while targeted forms of therapy exist that can be used for metastatic CRPC, we must continue to identify cancer-associated markers for the treatment of prostate cancers that have a resistance to hormone therapy and no androgen receptor (AR^neg^-AI prostate cancer).

Receptor Tyrosine Kinase like Orphan Receptor 1 (ROR1) is an oncofetal receptor tyrosine kinase (RTK)-like protein that is highly expressed during embryonic development but is minimally expressed in fully developed tissue [32,33]. However, it is upregulated in a wide variety of cancers, including prostate cancer [32,33,34,35,36,37,38,39]. ROR1 is known to promote cell growth, proliferation, and migration, which are cellular features that are important for embryogenesis [40,41]. While the underlying mechanism of ROR1 dysregulation remains unknown, emerging research suggests that the loss of certain microRNAs that normally repress ROR1 expression may be a possible mechanism of action that underlies ROR1 dysregulation [42,43,44,45]. As an RTK-like protein, ROR1 is known to activate many oncogenic pathways, including the central phosphatidylinositol 3-kinase (PI3K)-protein kinase B (AKT)-glycogen synthase kinase 3beta (GSK3β) cascade [32,33,34,35,36,37,38,46,47]. Numerous studies have shown that inhibiting ROR1 can effectively target cancer [33,39,48,49,50,51,52]. Many new tumor-associated markers are being explored at different pre-clinical and clinical stages of research that are targeted by a wide array of therapies, such as synthetically derived small molecule inhibitors, chemotherapy, and immunotherapy [53,54,55]. However, one untapped resource is the plethora of naturally derived compounds available from plant extracts. One such compound, known as 1,2,3,4,6 Penta-O-galloyl-β-D-glucose (PGG), has been shown to suppress metastasis and induce apoptosis in many cancers by downregulating malignant growth factor receptors and cytokine receptor signaling [56,57,58,59]. Recent evidence suggests that PGG may also act via the inhibition of ROR1, as shown through robust in silico ligand-protein docking models [60]. Our lab has previously investigated the anti-cancer effects of ROR1 inhibitor strictinin, which is a naturally derived compound that is very structurally similar to PGG [39,49,52]. Fultang et al. demonstrated through in silico analysis that strictinin and PGG bind to nearly the same domain on ROR1, but that PGG has a higher affinity for ROR1 than strictinin [49]. The study also utilized isothermal calorimetry experiments to verify that strictinin binds to ROR1 [49]. Therefore, there is significant evidence from prior research that suggests PGG is an inhibitor of ROR1 [49,60]. However, PGG has not been validated as an ROR1 inhibitor in any in vitro or in vivo cancer studies. 

In this study, we explore the mechanism by which PGG suppresses AR^neg^-AI PC3 prostate cancer cell survival and metastasis. Our results suggest that ROR1 is an important driver of cancer aggression in AR^neg^-AI prostate cancer that exhibits castration resistance and lacks a targetable AR. Our findings also suggest that the natural small molecule inhibitor PGG can affect ROR1 mediated AKT-GSK3β signaling and reduce cancer survival and metastasis. PGG can be further effective in reducing cancer cell viability when used along with docetaxel. The results from our experiments provide evidence for PGG to be an ROR1 inhibitor in prostate cancer cells and shows potential for the clinical treatment of AR^neg^-AI CRPC.

## 2. Results

### 2.1. ROR1 Levels Are Upregulated in AR^neg^-AI PC3 Cells 

**Figure 1:**
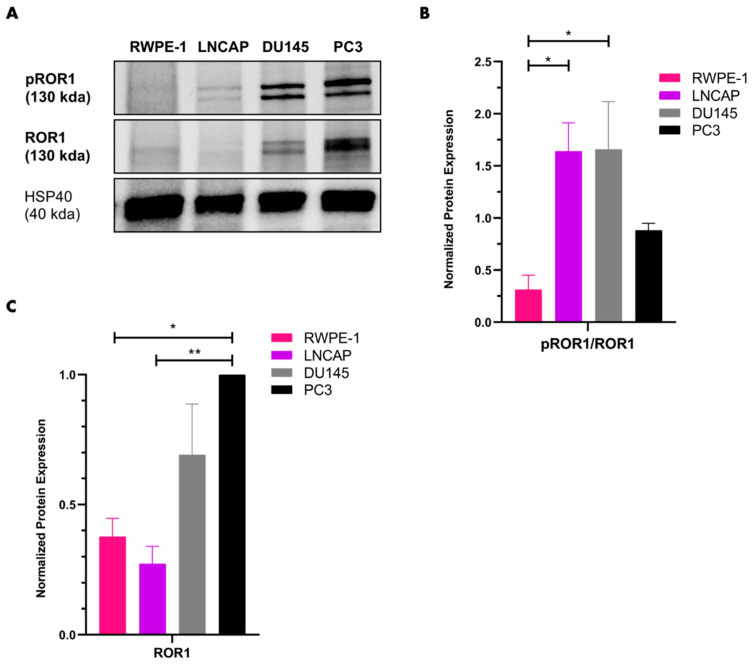
**ROR1 levels are elevated in aggressive prostate cancer cell lines.** (**A**) Representative western blot of phospho-ROR1 (Tyr786) and ROR1 in RWPE-1 (normal prostate epithelium), LNCAP (AR^pos^-AD), DU145 (AR^neg^-AI), and PC3 (AR^neg^-AI). (**B**) Quantification of immunoblot bands pROR1/ROR1 across different cell lines (all normalized to HSP40). Data are expressed as the mean +/− SEM from three independent western blots. (**C**) Quantification of immunoblot band ROR1 across different cell lines (all normalized to HSP40). Data are expressed as the mean +/− SEM from three independent western blots. [N = 3. ** = *p* value < 0.01; * = *p* value < 0.05].

To understand whether aggressive AR^neg^-AI prostate cancer cells exhibited upregulated levels of ROR1, immunoblots were performed on RWPE-1, LNCAP, DU145, and PC3 protein lysates. We noted an increase in the ratio of phospho-ROR1 (Tyr786) to total ROR1 in LNCAP and DU145 cells compared to PC3 and RWPE-1 (Figure 1A,B). However, it is important to note that RWPE-1 and LNCAP cells had lower levels of total ROR1 protein in comparison to DU145 and PC3 cells (Figure 1A,C). PC3 exhibited the highest expression of ROR1 (Figure 1A,C). These data suggest that ROR1 is highly expressed in the PC3 cell line and is moderately expressed in DU145 cells, while being minimally expressed in RWPE-1 and LNCAP cells.

### 2.2. PGG Induces Selective Lethality in PC3 Prostate Cancer Cells 

**Figure 2:**
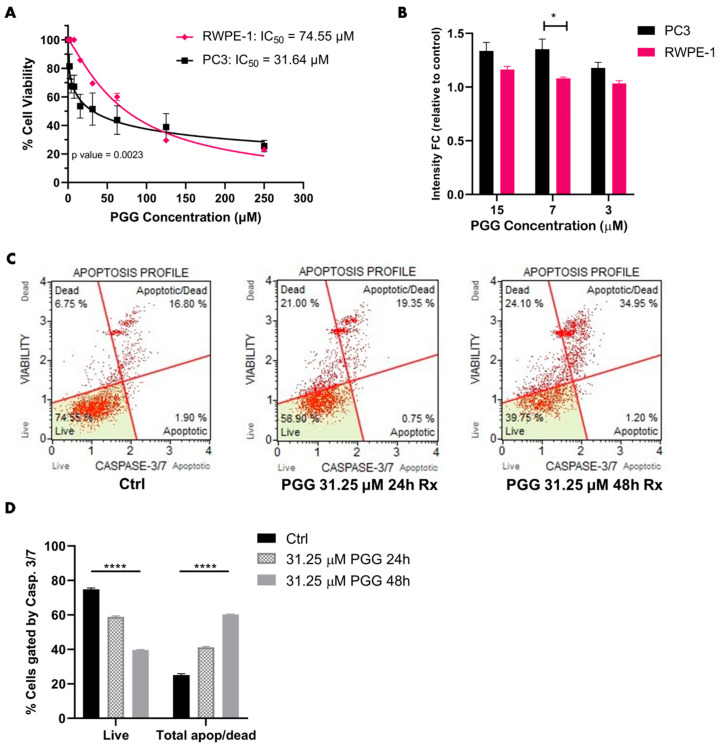
**PGG promotes apoptosis in PC3 cells.** (**A**) Cell viability of PC3 and RWPE-1 treated with PGG for 72 h. (**B**) Phosphatidylserine expression of PC3 and RWPE-1 cells after treatment with different PGG concentrations for 72 h. The data are represented as a fold change in the fluorescent intensity of Annexin V in PGG-treated cells compared to vehicle-treated cells. (**C**) Muse analyzer flow cytometry apoptosis profile of PC3 cells treated with vehicle, 31.25 μM PGG for 24 h, or 31.25 μM PGG for 48 h. Cells were sorted into live, apoptotic, apoptotic/dead, or dead categories by intensity of caspase 3 and caspase 7 expression. (**D**) Quantification of flow cytometry apoptosis profile on treated PC3 cells. [N ≥ 3. **** = *p* value < 0.0001; * = *p* value < 0.05].

**Figure 3:**
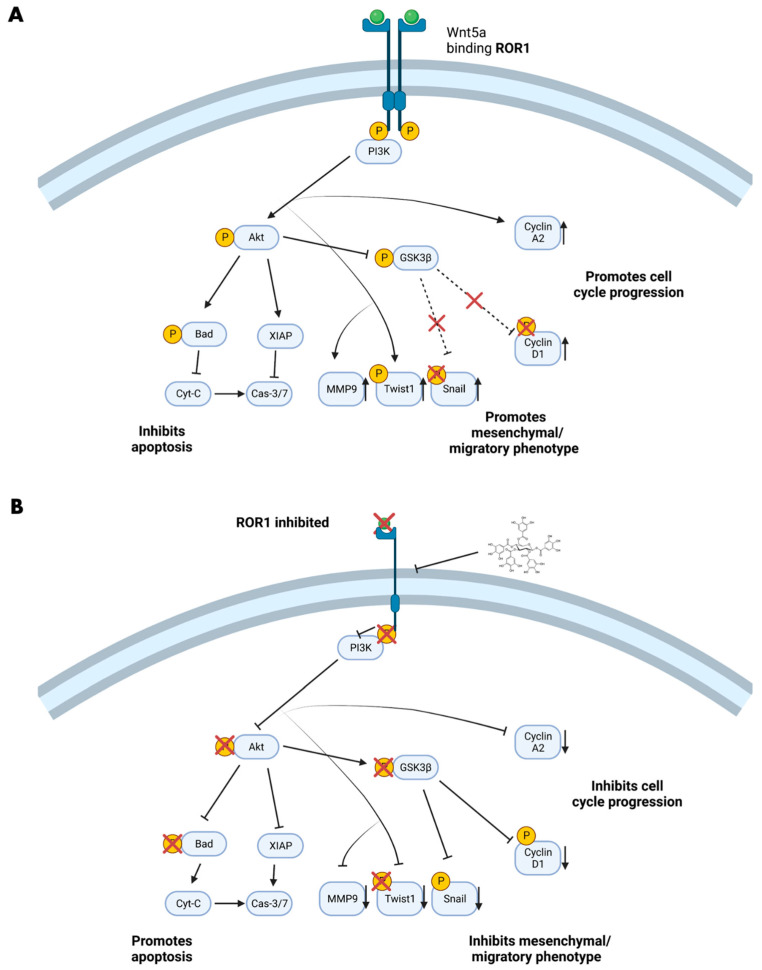
**The effects of PGG on the ROR1 molecular pathway.** (**A**) Depiction of active ROR1 and downstream signaling derived from the cited literature. Activated ROR1 leads to the phosphorylation of AKT and GSK3β. The phosphorylation of these proteins influences further downstream pro-cancerous signaling. (**B**) Depiction of inhibited ROR1 and downstream signaling based on the western blot experiments in PC3 cells and cited literature. PGG inhibits ROR1 and suppresses the phosphorylation of AKT and GSK3β, which causes a reduction in phospho-Bad, XIAP, MMP9, Twist1, Snail, Cyclin D1, and Cyclin A2 protein expression. The repression of these oncogenic signaling pathways promotes apoptosis and inhibits migration, invasion, and cell cycle progression in PC3 cells.

To establish an IC_50_ and identify whether PGG was cytotoxic to PC3 while minimally harming RWPE-1, both cell lines were treated with PGG doses ranging from 250 µM to 1.95 µM for 72 h. PGG displayed an IC_50_ of 31.64 µM in PC3, which was almost twofold lower than the IC_50_ of PGG in RWPE-1 (Figure 2A). Furthermore, an Annexin V phosphatidylserine expression assay exhibited an increase in the early apoptotic marker in PC3 cells treated with below IC_50_-level concentrations of PGG in comparison to RWPE-1 cells (Figure 2B). Since PGG was more cytotoxic and elicited more phosphatidylserine expression in PC3 cells in comparison to RWPE-1, we further assessed apoptosis in PC3 cells. We found that 24–48 h of PGG (31.25 µM) elicited significant cleaved caspase 3 and caspase 7 expression, which resulted in a substantial increase of PC3 cells that were categorized as apoptotic and dead in comparison to the control (Figure 2C,D). Together, these data strongly imply that PGG promotes cytotoxicity and apoptosis in PC3 cells (Figure 3B). Furthermore, the findings suggest that PGG more specifically targets highly aggressive AR^neg^-AI PC3 cells, while minimally targeting normal prostatic epithelial cells (RWPE-1). 

### 2.3. PGG Reduces ROR1 Levels and Leads to a Reduction in Oncogenic and Anti-Apoptotic Signaling in PC3 Cells

**Figure 4:**
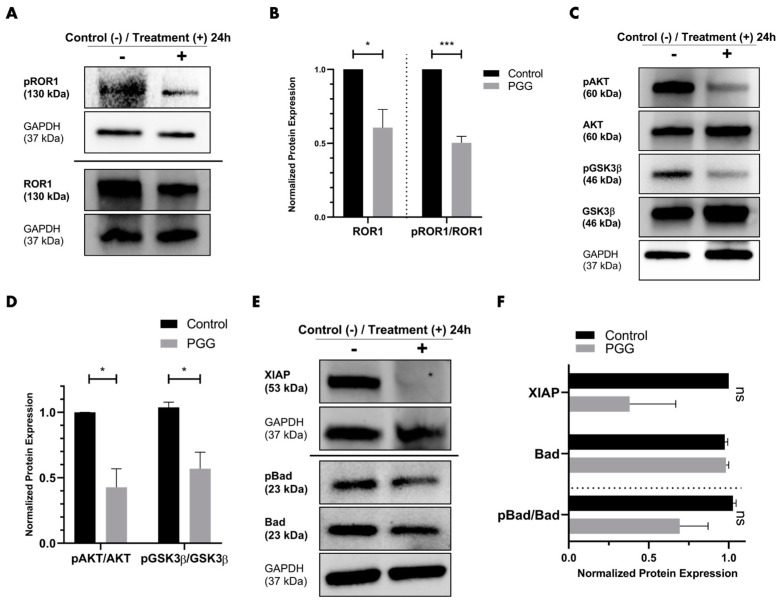
**PGG inhibits ROR1 intracellular domain phosphorylation, ROR1 expression, downstream oncogenic signaling, and anti-apoptotic signaling in PC3 cells.** (**A**) Representative western blot of phospho-ROR1 (Tyr786) and ROR1 on PC3 cells treated with vehicle or 31.25 μM PGG for 24 h. (**B**) Quantification of immunoblot bands phospho-ROR1 (Tyr786) and ROR1, all normalized to GAPDH. Data are expressed as the mean +/− SEM from three independent western blots. (**C**) Representative western blot of phospho-AKT (ser473), phospho-GSK3β (ser9), AKT, and GSK3β on PC3 cells treated with vehicle or 31.25 μM PGG for 24 h. (**D**) Quantification of immunoblot bands phospho-AKT (ser473), phospho-GSK3β (ser9), AKT, and GSK3β, all normalized to GAPDH. (**E**) Representative western blot of apoptotic markers XIAP, phospho-Bad (ser136), and Bad after treatment of PC3 cells with vehicle or 31.25 μM PGG for 24 h. (**F**) Quantification of immunoblot bands XIAP, phospho-Bad (ser136), and Bad, all normalized to GAPDH. Data are expressed as the mean +/− SEM from three independent western blots. [N = 3. *** = *p* value < 0.001; * = *p* value < 0.05; ns = not significant].

To evaluate whether PGG inhibited ROR1 in vitro, we treated PC3 cells with either vehicle or 31.25 µM of PGG for 24 h, followed by western blot analysis to assess ROR1, AKT, and GSK3β (Figure 4A,C). We opted to treat PC3 since these cells represent an aggressive and clinically difficult to treat AR^neg^-AI cancer that demonstrated the highest levels of ROR1 expression in comparison to normal prostatic epithelial cells and other prostate cancer cell lines such as DU145. Upon PGG treatment, we observed a reduction in phospho-ROR1 (Tyr786) and total ROR1 expression in comparison to the control (Figure 4A,B). Additionally, PC3 cells demonstrated a reduction in phospho-AKT (ser473) and phospho-GSK3β (ser9) upon treatment with PGG, as indicated by the phospho-AKT to total AKT ratio and phospho-GSK3β to total GSK3β ratio (Figure 4C,D). Because PGG inhibited phospho-AKT, we opted to evaluate whether PGG exerted an influence on phospho-B-cell lymphoma 2-associated agonist of cell death (BAD) and X-linked inhibitor of apoptosis (XIAP) as well. With PGG treatment, PC3 cells displayed a reduction in phospho-BAD (ser 136) and XIAP in comparison to the control (Figure 4E,F). Our data indicate that PGG is an inhibitor of ROR1 and can reduce the phosphorylation of its intracellular domain and overall protein expression (Figure 4B and Figure 3B). In doing so, PGG can also reduce downstream oncogenic signaling in PC3 cells, as observed by the reduction in phospho-AKT and phospho-GSK3β (Figure 4D and Figure 3B). PGG caused cytotoxicity and apoptosis in PC3 cells by reducing phospho-AKT, which inhibited downstream phospho-BAD and XIAP (Figure 4F and Figure 3B).

### 2.4. PGG Suppresses the Migration and Invasion of PC3 Cells While Minimally Impacting RWPE-1 Cells

**Figure 5:**
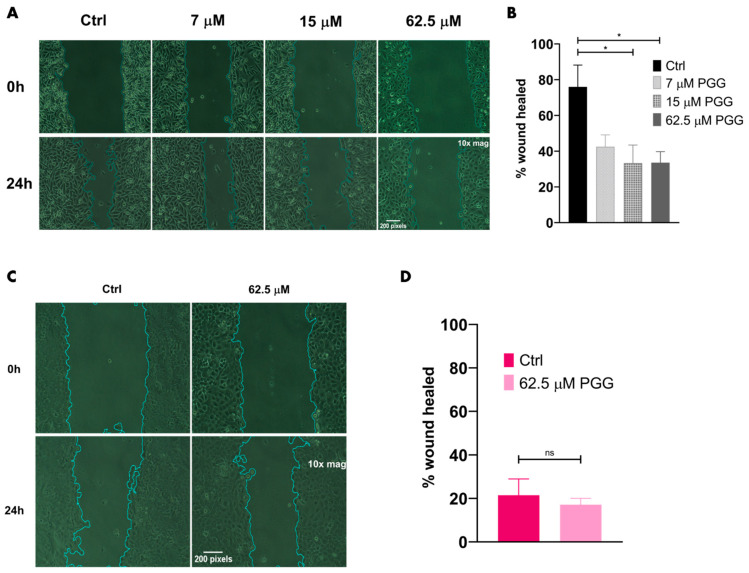
**PGG reduces the migration of PC3 cells with minimal impact on RWPE-1 migration.** (**A**) Wound healing assay to assess the migration of PC3 cells after treatment with vehicle, 7 µM PGG, 15 µM PGG, or 62.5 µM PGG for 24 h. (**B**) Quantification of the % wound healed to assess the migration of PC3 cells after 24-h vehicle or PGG treatment. (**C**) Wound healing assay to assess the migration of RWPE-1 cells after treatment with vehicle or 62.5 μM PGG for 24 h. (**D**) Quantification of the % wound healed to assess the migration of RWPE-1 cells after 24-h vehicle or PGG treatment. [N = 3. * = *p* value < 0.05; ns = not significant].

**Figure 6:**
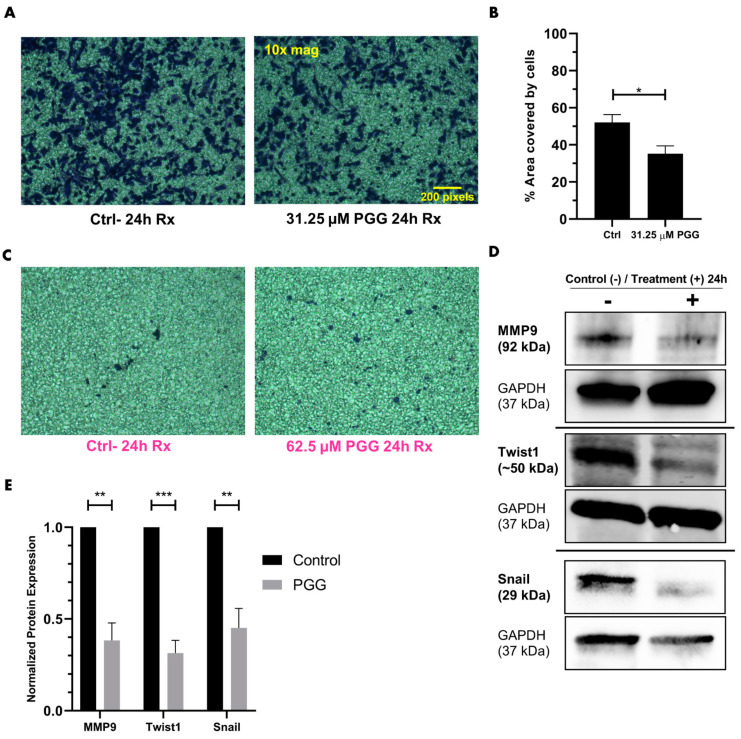
**PGG treatment suppresses the invasion of PC3 cells and inhibits the expression of migratory and invasive proteins downstream of the ROR1 molecular pathway.** (**A**) Boyden chamber assay to assess the invasion of PC3 cells through a basement membrane after treatment with vehicle or 31.25 μM PGG for 24 h. (**B**) Quantification of the % area covered by crystal violet stained cells to assess the invasion of PC3 cells after 24-h vehicle or PGG treatment. (**C**) Boyden chamber assay to assess the invasion of RWPE-1 cells after treatment with vehicle or 62.5 μM PGG for 24 h. (**D**) Representative western blot of MMP9, Twist1, and Snail on PC3 cells treated with vehicle or 31.25 μM PGG for 24 h. (**E**) Quantification of MMP9, Twist1, and Snail immunoblot bands normalized to GAPDH. Data are expressed as the mean +/− SEM from three independent western blots. [N = 3. *** = *p* value < 0.001; ** = *p* value < 0.01; * = *p* value < 0.05].

To understand whether PGG impacted prostate cancer cell migration and invasion, PC3 cells were subjected to migratory studies over a period of 24 h with 7 µM PGG, 15 µM PGG, 62.5 µM PGG, or control (DMSO) treatment. Invasion assays were performed over 24 h with 31.25 µM PGG or control treatment. PGG was found to substantially reduce the migration of PC3 cells at 15 µM and inhibited the invasion of PC3 cells at 31.25 µM (Figure 5A,B and Figure 6A,B). The migration and invasion of RWPE-1 cells were also evaluated over a period of 24 h with 62.5 µM PGG or control (DMSO) treatment. While PGG significantly inhibited the migration of PC3 cells at 62.5 µM, PGG had no significant impact on the migratory and invasive phenotype of RWPE-1 cells at the high dose of 62.5 µM (Figure 5A–D and Figure 6C). Additionally, we treated PC3 and RWPE-1 with up to 125 µM of PGG and found that cell viability was not significantly impacted within a 24-h period, which further corroborates the observed suppression of migration and invasion in PC3 cells treated with PGG (Figure S1). We predicted that the reduction of PC3 migration and invasion may be due to changes in downstream molecular signaling. Therefore, to further corroborate the phenotypic results observed, we assessed changes to downstream EMT markers in PC3 cells after treatment with a near-IC_50_ dose of PGG. We identified that PC3 cells treated with PGG exhibited markedly lower levels of matrix metalloproteinase-9 (MMP9), Snail, and Twist1 in comparison to control treatments (Figure 6D,E). Together, these findings suggest that PGG selectively inhibits the migration and invasion of PC3 cells while minimally impacting RWPE-1 cells, which occurs due to PGG’s ability to inhibit the ROR1-mediated AKT-GSK3β pathway and subsequently suppress mesenchymal proteins Twist1, Snail, and MMP9 in PC3 cells. ROR1 inhibition may cause a reversal in EMT, known as mesenchymal-to-epithelial transition (MET), which potentially underscores the reduction in migration, invasion, and mesenchymal protein expression seen in PGG-treated PC3 cells.

### 2.5. PGG Causes S Phase Cell Cycle Arrest in PC3 Cells

**Figure 7:**
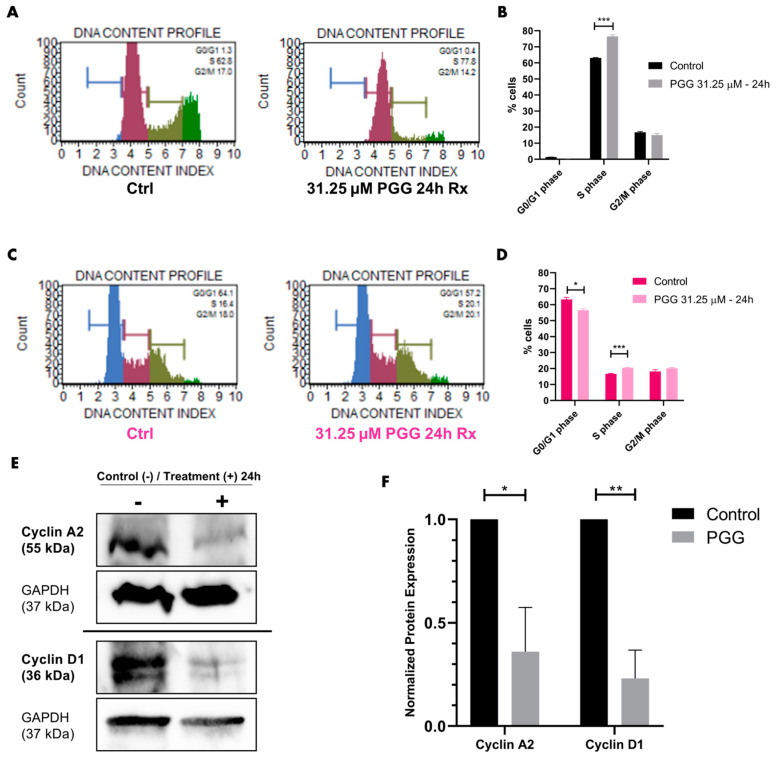
**PGG arrests PC3 cells in S phase and reduces Cyclin D1 and Cyclin A2 expression.** (**A**) Muse analyzer flow cytometry of PC3 cells treated with vehicle or 31.25 μM PGG for 24 h. Cells were sorted into different cell cycle phases by propidium iodide-based staining of DNA. (**B**) Quantification of cell cycle flow cytometry on treated PC3 cells. (**C**) Muse analyzer flow cytometry of RWPE-1 cells treated with vehicle or 31.25 μM PGG for 24 h. Cells were sorted into different cell cycle phases by propidium iodide-based staining of DNA. (**D**) Quantification of cell cycle flow cytometry on treated RWPE-1 cells. (**E**) Immunoblot of Cyclin A2 and Cyclin D1 on PC3 cells treated with vehicle or 31.25 μM PGG for 24 h. (**F**) Quantification of Cyclin A2 and Cyclin D1 immunoblot bands (normalized to GAPDH). Data are expressed as the mean +/− SEM from three independent western blots. [N = 3. *** = *p* value < 0.001; ** = *p* value < 0.01; * = *p* value < 0.05].

Cyclins are important for the progression of the cell cycle. Because PI3K-AKT-GSK3β signaling impacts Cyclin D1 and Cyclin A2 dynamics, we chose to determine whether ROR1-PI3K-AKT-GSK3β inhibition by PGG caused cell cycle arrest and changes to Cyclin D1 and Cyclin A2 expression. PC3 cells that were treated for 24 h with 31.25 μM of PGG exhibited S phase cell cycle arrest in comparison to non-treated PC3 cells (Figure 7A,B). RWPE-1 also exhibited a statistically significant S phase cell cycle arrest upon 31.25 μM PGG treatment, but the arrest was smaller in magnitude in comparison to PC3 cells treated with PGG (Figure 7C,D). Because PGG caused a larger magnitude of S-phase arrest in PC3 cells versus RWPE-1, we further assessed cell cycle signaling downstream of the PI3K-AKT-GSK3β axis. Our findings demonstrated that PGG caused a reduction in Cyclin D1 and Cyclin A2 expression in PC3 cells when compared to the control treatment (Figure 7E,F). In summary, our data suggest that PGG specifically inhibits PC3 cell cycle progression, in the form of an S phase arrest, by downregulating Cyclin D1 and Cyclin A2.

### 2.6. The Combination of PGG and Docetaxel Synergistically Decreases PC3 Cell Viability

**Figure 8:**
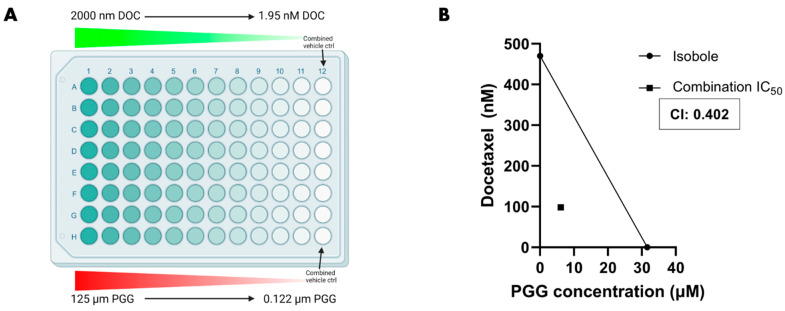
**The combination of PGG and docetaxel is synergistic and lowers the IC_50_ of both compounds in PC3 cells.** (**A**) Depiction of PC3 cells in a 96-well plate treated with Docetaxel and PGG. Cells were treated with the highest concentration of docetaxel and PGG and serially diluted to the lowest combo-concentration. Cell viability was compared to a control lane to assess % cell viability. (**B**) The isobologram is represented with a line drawn from the IC_50_ of docetaxel alone (y-intercept) to the IC_50_ of PGG alone (x-intercept). The combination IC_50_ is represented by the square dot and was used to calculate the CI (combination index) = 0.402. The IC_50_ values were obtained from cell viability experiments. For more information on IC_50_ values, refer to Figure S2. [N ≥ 3].

We sought to identify whether PGG and docetaxel combination therapy would display a synergistic effect on PC3 cells in vitro. To determine the IC_50_ of both compounds when used together, PC3 cells were treated with a range of both PGG and docetaxel concentrations for 72 h, followed by an assessment of cell viability (Figure 8A). From this experiment, we noted that the IC_50_ of PGG and docetaxel decreased about five-fold for both compounds when used in combination, with a combination index (CI) of 0.402 (Figure 8B and Figure S2). Additionally, there was a left shift in the combo-therapy cell viability curve in comparison to the monotherapy curve when cell viability was plotted against the PGG concentration and docetaxel concentration (Figure S2). The combination IC_50_ was also below the isobole (Figure 8B). The five-fold reduction in the IC_50_ of both compounds, isobologram analysis, and a CI of less than 1 all indicate that PGG and docetaxel act in synergy to lower the concentration of both compounds needed for a 50% cytotoxic effect [61,62]. PGG may be used in combination with docetaxel to lower the amount of both compounds required to generate a lethal response in PC3 cells. Furthermore, this combination therapy could potentially increase the specificity of treatment for cancers expressing ROR1. 

### 2.7. DU145 Cells Have a Modest Phenotypic Response to PGG Treatment

**Figure 9:**
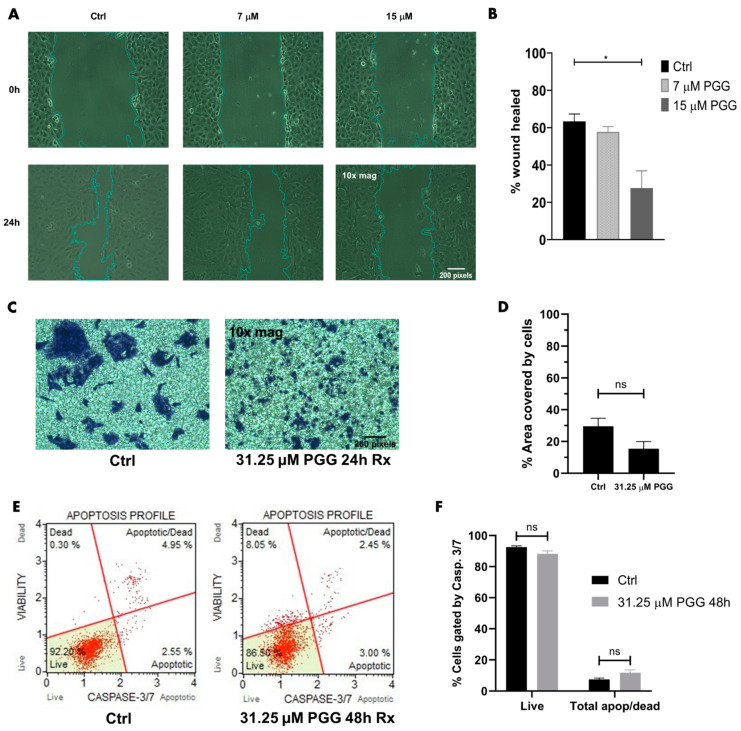
**PGG exhibits anti-migratory and anti-invasive effects on DU145 cells.** (**A**) Wound healing assay to assess the migration of DU145 cells after treatment with vehicle, 7 μM PGG, or 15 μM PGG for 24 h. (**B**) Quantification of the % wound healed to assess the migration of DU145 cells after 24-h vehicle or PGG treatment. (**C**) Boyden chamber assay to assess the invasion of DU145 cells through a basement membrane after treatment with vehicle or 31.25 μM PGG for 24 h. (**D**) Quantification of the % area covered by crystal violet stained cells to assess the invasion of DU145 cells after 24-h vehicle or PGG treatment. (**E**) Representative data of Muse analyzer flow cytometry apoptosis profile of DU145 cells treated with vehicle or 31.25 μM PGG for 48 h. Cells were sorted into live, apoptotic, apoptotic/dead, or dead categories by intensity of caspase 3 and 7 expression. (**F**) Quantification of flow cytometry apoptosis profile on treated DU145 cells. [N ≥ 3. * = *p* value < 0.05; ns = not significant].

We previously identified that DU145 cells, another AR^neg^-AI prostate cancer, have a moderate expression of ROR1 compared to PC3 cells (Figure 1). Hence, we wanted to determine whether PGG decreased DU145 cell aggression. Experimentation revealed that PGG treatment decreased the migration and invasion of DU145 cells (Figure 9A–D). However, PGG did not cause a significant increase in pro-apoptotic caspase 3/7 expression and did not significantly increase apoptosis of DU145 cells in comparison to the control (Figure 9E,F). Together, these data indicate that PGG reduces DU145 cell migration and invasion but is not as lethal to DU145 as it is to PC3.

## 3. Discussion

Metastatic CRPC, which can display aggressive survival and metastatic characteristics independent of the androgen signaling axis, is currently treated with hormone therapy, chemotherapy, and forms of targeted therapy such as sipuleucel-T, Lutetium-177-PSMA-617, pembrolizumab, and PARP inhibitors [1,2,3,4,10,11,12]. The chemotherapeutic agents used to treat metastatic CRPC can often cause adverse effects arising from toxicity to normal cells, which can lead to the discontinuation of therapy [29,30,31]. Furthermore, AR^neg^-AI prostate cancer is a subtype of CRPC that has more aggressive characteristics than AR^pos^-AI prostate cancer and is increasing in incidence [4,6,7,8,9]. Obstacles such as toxicity-induced adverse events from chemotherapy and AR^neg^ castration resistance require the need to identify more cancer-specific molecular targets that would allow for effective treatment of AR^neg^-AI prostate cancer while minimally harming normal tissue. 

One emerging cancer-associated target is Receptor tyrosine kinase like Orphan Receptor 1 (ROR1), which is an oncofetal RTK-like protein that is highly expressed in embryogenesis and minimally expressed in developed tissue [32,33]. However, for reasons currently unknown, ROR1 is uniquely upregulated in many cancers, including prostate cancer [32,33,34,35,36,37,38]. ROR1 can be a powerful target due to its specific upregulation in cancer. Therefore, this study investigates how the targeting of ROR1-mediated oncogenic signaling exhibits selective lethality of highly aggressive AR^neg^-AI PC3 cells, moderate lethality of AR^neg^-AI DU145 cells, and minimal lethality of normal prostatic epithelial cell line RWPE-1. This study identifies pentagalloyl glucose (PGG), for the first time, as an ROR1 inhibitor that targets ROR1-expressing PC3 cancer cells in vitro. We also identify that PGG leads to the inhibition of the central PI3K-AKT-GSK3β pathway and abrogates even further downstream signaling related to anti-apoptosis, EMT, and cell cycle progression in PC3 cells.

First, we identified that ROR1 expression was most elevated in PC3 in comparison to the other cell lines (Figure 1). Since prior research established PGG as an ROR1 inhibitor, we wanted to explore whether PGG exhibited lethality against PC3 cells that express high levels of ROR1 [60]. We found that PGG was selectively cytotoxic to PC3 cells and induced apoptosis of PC3 in comparison to RWPE-1 cells as determined by MTT proliferation and Annexin V fluorescent expression assays (Figure 2A,B). The IC_50_ of PGG (31.64 µM) was almost two-fold lower in PC3 compared to RWPE-1 (74.55 µM) (Figure 2A). PGG increased phosphatidylserine expression in PC3 cells in comparison to RWPE-1 cells at concentrations lower than the IC_50_, which demonstrates that PGG initiates apoptosis of PC3 cells at relatively low concentrations (Figure 2B). Furthermore, 24–48 h of 31.25 µM PGG induced a marked increase in pro-apoptotic caspase 3 and caspase 7 expression as measured by flow cytometry, which underscored the increase in categorization of PGG-treated PC3 cells as apoptotic and dead in comparison to the control (Figure 2C,D). 

To further validate PGG as an ROR1 inhibitor and assess the molecular mechanism by which PGG selectively inhibits PC3, we corroborated prior in silico analysis with robust in vitro experiments [60]. ROR1 becomes activated upon ligand binding, which triggers phosphorylation of the intracellular domain of ROR1, homodimerization, and subsequent downstream oncogenic PI3K-AKT-GSK3β signaling [33,35,37,38,48,63]. Increased phosphorylation of AKT (ser473) and GSK3β (ser9) is often observed in dysregulated PI3K-AKT-GSK3β signaling that arises from aberrant RTK activity (Figure 3A) [63,64]. Because ROR1 is highly expressed in PC3 cells, we conducted 24-h PGG treatments in PC3 and found that PGG suppressed phosphorylation of the intracellular domain of ROR1 (Tyr786) and overall ROR1 expression (Figure 4A,B). PGG also led to a reduction in downstream phospho-AKT and phospho-GSK3β in PC3 cells (Figure 4B–D). The inhibition of phospho-ROR1 (Tyr786) and overall ROR1 expression by PGG may indicate that the compound competitively inhibits wnt5a and other endogenous ligands from binding to and activating the ROR1 receptor [65]. 

It is well known that phospho-AKT (active) can phosphorylate downstream BAD and increase XIAP expression, leading to an inhibition of the apoptotic cascade (Figure 3A) [66,67,68,69]. In particular, the phosphorylation of BAD sequesters the protein away from the anti-apoptotic proteins B-cell lymphoma extra-large (BCL-xL) and B-cell lymphoma 2 (BCL2) and allows them to suppress apoptosis (Figure 3A) [66,69]. XIAP directly interacts with and inhibits caspase 3 and caspase 7 (Figure 3A) [67,68]. Since PGG induced significant apoptosis of PC3 cells, we wanted to explore the underlying molecular mechanism behind this phenotypic change. We were able to determine that PGG induced significant apoptosis in PC3 cells due to a reduction in phospho-AKT, phospho-BAD, and XIAP downstream of ROR1, allowing for the initiation and execution of the pro-apoptotic pathway (Figure 4C–F and Figure 3B). 

The central oncogenic PI3K-AKT-GSK3β pathway affects more signaling modalities in addition to anti-apoptotic pathways. Proteins such as Twist1, Snail, and MMP9 are implicated in the EMT, migration, and invasion of cancer [70,71,72]. It is known that the malignant PI3K-AKT cascade increases the phosphorylation and expression of downstream Twist1 [73,74,75]. Furthermore, GS3kβ phosphorylates Snail and decreases its stability via ubiquitin–proteasome degradation [70,71,75,76]. Cells exhibiting oncogenic PI3K-AKT signaling will suppress the function of GSK3β by phosphorylation, which allows for the stable expression of non-phosphorylated Snail [70,71,75]. The PI3K-AKT-GSK3β pathway also increases the expression of a MMP9 [72,77,78,79]. Snail and Twist1 suppress E-cadherin (adherens junctions) and zonula occludens-1 (ZO-1) (tight junction) expression, which are important adhesion molecules in cells that exhibit an epithelial/non-migratory phenotype [80,81,82]. Additionally, Twist1 and Snail increase the expression of other mesenchymal proteins that are important for migration and invasion [83,84,85]. Finally, Twist1 and Snail are known to increase the stemness of cancer cells, another important process that enables cancer cells to self-renew, resist chemotherapy, and metastasize [83,84,85]. Hence, Snail and Twist1 are crucial proteins that influence EMT and cancer stem cell (CSC) formation, two processes that have much overlap and lead to heightened cancer aggression and metastasis [83,84,85]. Another marker that was probed post-PGG treatment was MMP9, which increases the cell’s ability to degrade the extracellular matrix for invasion and migration [72]. Our data demonstrated that PGG significantly impacted the migration and invasion of highly metastatic PC3 cells, while minimally impacting the phenotype of non-metastatic RWPE-1 cells (Figure 5A–D and Figure 6A–C). These results were further corroborated by western blot analysis that revealed a reduction in Twist1, Snail, and MMP9 in PC3 cells treated with PGG in comparison to control treatments (Figure 6D,E and Figure 3B). These experiments may suggest that PGG, by inhibiting the ROR1-AKT-GSK3β cascade and metastatic proteins, potentially causes a mesenchymal-to-epithelial transition that reduces migration, invasion, and possibly the stemness of PC3 cells.

The PI3K-AKT-GSK3β pathway also influences the cell cycle. PI3K-AKT signaling is known to enhance Cyclin D1 and Cyclin A2 expression, which influences progression into the S phase and G2/M phase, respectively (Figure 3A) [86,87,88,89,90,91]. Therefore, we wanted to understand whether PGG caused cell cycle arrest and changes to Cyclin D1 and Cyclin A2 expression. Our flow cytometry data revealed that PC3 cells were arrested in S phase upon treatment with PGG (Figure 7A,B). This was supported by a reduction in Cyclin D1 and Cyclin A2 expression when PC3 cells were treated with 31.25 µM PGG (Figure 7E,F and Figure 3B). RWPE-1 cells were only minimally impacted by PGG and had a slight increase in S phase arrest upon PGG (31.25 µM) treatment in comparison to PC3 cells (Figure 7C,D). Overall, PGG significantly decreases Cyclin D1 and Cyclin A2 expression, which arrests PC3 cells in the S phase of the cell cycle.

Chemotherapeutic agents used to treat prostate cancer (e.g., docetaxel, cabazitaxel) are known to generate significant adverse effects, especially in patients who must undergo aggressive chemotherapy regimens [29,30,31,92]. Docetaxel is a current standard chemotherapeutic agent for metastatic CRPC [28]. Though PGG generated a specific cytotoxic, cell cycle arrest, anti-migratory, and anti-invasive effect in PC3 cells, we wanted to investigate whether PGG and docetaxel could be used in lower amounts to induce significant cytotoxicity when used in combination. Therefore, we examined the cytotoxic effect of combination PGG and docetaxel therapy in vitro (Figure 8A). Experimentation in PC3 cells revealed that PGG and docetaxel together were synergistic and lowered the IC_50_ of both compounds (Figure 8B and Figure S2). In clinical terms, this indicates that using a combination of PGG and docetaxel may lower the concentration of both compounds required to achieve a desired therapeutic effect. The combination of PGG and docetaxel may lower the adverse effects of docetaxel, while increasing the specificity of therapy through PGG-induced inhibition of ROR1. This finding shows promise for patients who may have to undergo aggressive chemotherapy and have metastatic CRPC that expresses high levels of ROR1. Evaluating the effects of PGG in combination with other standard prostate cancer treatments (cisplatin, cabazitaxel, PARP inhibitors, pembrolizumab, etc.), which was not attempted in this study, should be performed in future studies.

DU145, an AR^neg^-AI prostate cancer cell line, exhibited moderate levels of ROR1 expression (Figure 1). In order to compare the efficacy of PGG in PC3 vs. DU145, we used near-IC_50_ values of PGG in PC3 cells, such as 15 µM and 31.25 µM, to treat DU145 cells. We found that PGG treatment inhibited the migration and invasion of DU145, while not significantly causing apoptosis (Figure 9). Hence, we concluded that PGG exhibits anti-cancer properties in DU145 cells but may not be as potent in DU145 when compared to PC3 cells. This may be due to DU145 cells exhibiting lower levels of ROR1 expression in comparison to PC3 cells (Figure 1A,C). This implies that PGG may still be an effective form of therapy for prostate cancers that have phenotypic and molecular features like DU145 cells, especially if the goal is to reduce the metastatic potential of the cancer. Perhaps, a slightly higher concentration of PGG or a combination of PGG and docetaxel can induce further anti-migratory, anti-invasive, and pro-apoptotic effects on DU145 cells. Future studies should explore the effects of combining PGG and docetaxel, as well as other combinations with standard anti-prostate cancer agents, on DU145 cells. Given the lack of targeted markers for AR^neg^-AI prostate cancer, ROR1 inhibition may still be a viable option for clinical prostate cancers that exhibit the characteristics of DU145 cells. 

In this study, we have identified the importance of ROR1 as a driver of cancer aggression in AR^neg^-AI prostate cancer, which is a form of prostate cancer that exhibits castration resistance and lacks a targetable AR. Additionally, our findings suggest that the small molecule ROR1 inhibitor, PGG, selectively targets ROR1-expressing cancer cells and suppresses proliferation, anti-apoptosis, migration, invasion, and cell cycle progression. Moreover, PGG and docetaxel can be used at significantly lower amounts to elicit cytotoxicity of PC3 cells when used in combination. We also unravel changes to far downstream signaling pathways in PC3 cells that are influenced by the ROR1-AKT-GSK3β axis, including the anti-apoptosis, EMT, migratory, invasive, and cell cycle signaling cascades that were suppressed by PGG. It is important to note that while we characterize the anti-cancer properties of PGG through its ability to modulate ROR1-mediated AKT-GSK3β signaling based on prior literature and our results, it is possible that PGG can also exhibit these effects by impacting other cancer-associated markers. This warrants further research. ROR1 has great potential as an emerging cancer-associated molecular target that can be used to subtype and treat prostate cancer. Furthermore, the ROR1 inhibitor PGG has great potential for the clinical treatment of AR^neg^-AI CRPC. 

ROR1 inhibition for the treatment of cancer is emerging as a potential therapeutic strategy. ROR1 has been demonstrated to be an important cancer-associated marker for chronic lymphocytic leukemia (CLL) [93]. CLL patients with high ROR1 expression levels have a shorter median overall survival than CLL patients with low ROR1 expression [93]. Small molecule ROR1 inhibitors (KAN0439834, KAN0441571C) have been shown to induce apoptosis of CLL cells in culture [94,95]. Furthermore, the use of an ROR1 monoclonal antibody (cirmtuzumab) is being studied in clinical trials as a potential treatment option for CLL [96,97,98]. A new clinical trial will soon be investigating the combination of cirmtuzumab and docetaxel for the treatment of metastatic CRPC [99]. Through our experiments, we have established PGG as an ROR1 inhibitor that exerts significant anti-cancer effects against AR^neg^-AI prostate cancer cells. The findings in this study warrant further research that explores whether PGG can be a potent ROR1 inhibitor that can effectively treat metastatic CRPC in vivo, and whether the combination of PGG and docetaxel enhances in vivo treatment efficacy. 

## 4. Materials and Methods

### 4.1. Cell Culture Techniques

PC3 (ATCC CRL-1435), RWPE-1 (ATCC CRL-11609), LNCAP (ATCC CRL-1740), and DU145 (HTB-81) cells were maintained in culture medium and subcultured as per American Type Culture Collection (ATCC) recommendations. LNCAP cells were grown in RPMI-1640 medium that was supplemented with 10% fetal bovine serum. For more details regarding the other cell lines, see Sivaganesh et al. [52]. 

### 4.2. Preparation of PGG and Vehicle Control for Cell Treatment

Penta-O-galloyl-β-D-glucose hydrate (Sigma, USA, #G7548), referred to as PGG, was obtained at 25 mg and dissolved in DMSO to generate a 10 mg/mL and 100 mg/mL stock solution. By using the molecular weight of PGG, we calculated the volume of the compound to be mixed with a certain volume of media to generate the desired concentration (up to 250 µM). DMSO alone was dissolved in media to serve as the vehicle control. The DMSO concentration did not exceed 0.5% of the cell culture media.

### 4.3. Preparation of Docetaxel and Vehicle Control for Cell Treatment

Docetaxel (Sigma, New York, NY, USA, #01885) was obtained as a 5 mg powder and dissolved in DMSO to generate a stock solution. By using the molecular weight of docetaxel, we calculated the volume of the drug to be mixed with a certain volume of media to generate the desired concentration (up to 2500 nM). To make the control media solution, DMSO was dissolved in media to match the percentage of DMSO in conditions treated with the highest concentration of docetaxel. The DMSO concentration did not exceed 0.5% of the cell culture media.

### 4.4. Immunoblot

PC3 cells were grown in cell culture flasks, then treated with 31.25 µM PGG or vehicle control. RWPE-1, DU145, and LNCAP cells were also grown in cell culture flasks for western blot experiments. After 24 to 48 h of incubation in 37 °C, cells were gently scraped within ice cold 1x PBS and centrifuged to isolate the cell pellets. The cells were lysed using RIPA buffer (pH 8.0, 1% Triton-X-100, 0.1% SDS, 50 mM Tris, 150 mM NaCl, 0.5% sodium deoxycholate) containing 1x protease inhibitor and 1x ethylenediaminetetraacetic acid (EDTA), then centrifuged at 14,000× *g* for 15 min to collect the supernatant that contained the protein. Protein concentrations were determined using the Pierce 660 nm Protein Assay (Thermofisher, Waltham, MA, USA, #22660). The proteins were diluted in a 2x Laemmli buffer at a 1:1 ratio and denatured by heating the samples at 100 °C for 5–10 min. Equal amounts of protein (15–30 µg) were loaded into the wells of a Tris-glycine gel (4–15%), followed by protein separation at 100 V for 85 min. The separated proteins on the gel were transferred to a nitrocellulose membrane using the iBlot dry transfer instrument (Thermofisher). Then, the proteins of interest were probed using a 1:1000 dilution of primary antibodies and a 1:3000 dilution of HRP-conjugated secondary antibodies or 1:15,000 dilution of Licor IRDye secondary antibodies that corresponded to the species within which the primary antibody was produced; diluents were prepared in the form of 5% BSA or 5% milk in 1x Tris-buffered saline with tween (TBST). A Biorad chemiluminescence imager was used to image membranes incubated with HRP-conjugated secondary antibodies and luminol detection reagent. The Licor Odyssey DLx system was used to image membranes incubated with Licor IRDye secondary antibodies. Captured bands were quantified and normalized to housekeeping proteins GAPDH or HSP40 using ImageJ software (https://imagej.net/ij/, accessed on 8 May 2024).

### 4.5. Antibody List for Western Blot

ROR1 (Cell signaling, USA, #16540S); pROR1-Tyr786 (Thermofisher, USA, #PA5-64807); p-AKT-ser473 (Cell signaling, Danvers, MA, USA, #4060S); AKT (Cell signaling, USA, #9272S); p-GSK3β-ser9 (Cell signaling, USA, #5558S); GSK3β (Cell signaling, USA, #12456S); XIAP (Cell signaling, USA, #14334S); Bad (Cell signaling, USA, #9239S); p-Bad-ser136 (Cell signaling, USA, #4366S); Snail (Cell signaling, USA, #3895S); Twist1 (Cell signaling, USA, #69366S); MMP-9 (Cell signaling, USA, #13667S); Cyclin A2 (Cell signaling, USA, #4656S); Cyclin D1 (Cell signaling, USA, #2978S); GAPDH (scbt, Santa Cruz, CA, USA, #C2819); HSP40 (Cell signaling, USA, #4868S); IRDye 800CW Goat anti-rabbit secondary (Licor, Lincoln, NE, USA, #926-32211); IRDye 800CW Goat anti-mouse secondary (Licor, USA, #926-32210); HRP conjugated Goat anti-mouse secondary (Biorad, Hercules, CA, USA, #1706516); HRP conjugated Goat anti-rabbit secondary (Cell signaling, USA, #7074S).

### 4.6. Cell Viability Assay (MTT)

First, PC3 and RWPE-1 cells were seeded in 96-well plates at equal densities per well (e.g., 10,000 cells/well). After the cells attached, which normally occurred by day 2, PC3 and RWPE-1 were treated with PGG doses ranging from 250 µM to 1.95 µM for 24–72 h. Then, the PGG and media mixture were aspirated and cells were replenished with fresh media and MTT dye. After 2–4 h of incubation and subsequent addition of DMSO, the absorbance values of the wells were obtained by reading the plates at a 540 nm wavelength. Cell viability was reported as the absorbance from treated wells to non-treatment or vehicle control wells. 

### 4.7. Phosphatidylserine Apoptosis Assay

PC3 and RWPE-1 cells were plated at equal densities (25,000–50,000) per well in a clear bottom, black walled, 96-well plate (Corning, New York, NY, USA, #3904). Following 72 h of treatment with different concentrations of PGG or vehicle control, cells were treated with a mixture of fresh media and orange fluorescent apopxin at a 1:1 ratio. Apopxin working solution was made as per protocol instructions (AATbio, Pleasanton, CA, USA, #22794). The plate was incubated for 1 h. Then, fluorescence intensities were read using a Tecan microplate reader at an excitation of 540 nm and an emission of 590 nm. Raw intensity values represented the amount of cell surface phosphatidylserine present.

### 4.8. Caspase 3/7 Apoptosis Flow Cytometry

PC3 and DU145 cells were plated in 6-well dishes at equal densities per well and treated with different concentrations of PGG or vehicle control for a maximum of 48 h. Then, the cells underwent preparation for flow cytometry using the Muse Caspase 3/7 kit (Luminex, Austin, TX, USA, #MCH100108). Samples were run through a Muse flow cytometer, which provided the percentage of cells at different stages of apoptosis. Approximately 2000 cells were analyzed per run. 

### 4.9. Wound Healing Assay

Cells were seeded and grown to near 100% confluence within 6-well plates. Then, scratches were generated using a pipette tip and either PGG or vehicle control was introduced into the wells. Cells that were detached upon generating the scratches were rinsed off prior to introducing the treatment. At the 0 h and 24 h mark, bright field images of the scratches were taken (at 10× magnification) for each condition using an inverted phase contrast microscope. The wound sizes at the 0 h and 24 h mark were quantified using ImageJ and were used to calculate the percentage of the wound healed.

### 4.10. Invasion Assay

A Boyden chamber consists of an insert that sits within the wells of a 24-well plate. The insert is normally placed on top of media within the well. The top chamber is the insert, with the basement membrane being the barrier between the top chamber and bottom chamber. The bottom chamber is the well that contains the insert. After 24 h of serum starvation, cells were seeded into the top chamber in serum-starved media containing PGG or vehicle control. The bottom chamber contained media fully supplemented with serum that acted as a chemoattractant. Over a period of 24 h, cells invaded through the basement membrane and migrated to the other side of the membrane that was submerged in the bottom chamber’s media. After allowing the cells to invade and migrate for 24 h, the cells attached to the basement membrane on the bottom chamber side were stained with crystal violet, imaged with an inverted phase contrast microscope (at 10× magnification), and quantified using ImageJ. 

### 4.11. Cell Cycle Flow Cytometry

PC3 and RWPE-1 cells were plated in 6-well plates at equal densities per well and treated with different concentrations of PGG or vehicle control for 24 h. Then, the cells underwent preparation for flow cytometry using the Muse Cell Cycle kit (Luminex, USA, #MCH100106). Samples were run through a Muse flow cytometer, which provided the percentage of cells at different stages of the cell cycle. 

### 4.12. Combination Drug Treatment MTT Proliferation Assay

PC3 cells were plated at the same cell density per well. Then, after the cells attached on day 2, the 96-well plate was treated with serial dilutions of PGG and docetaxel for 72 h. Then, the compound and media mixture were aspirated and cells were replenished with fresh media and MTT dye. After 2–4 h of incubation and subsequent addition of DMSO, the absorbance values of the wells were obtained by reading the plate at a wavelength of 540 nm. Cell viability was reported as the absorbance from treated wells to non-treatment or vehicle control wells. Synergism was determined by isobologram analysis and identifying a statistically significant change in the IC_50_ when comparing combination therapy to monotherapy [61,62]. Furthermore, a combination index (CI) was calculated and a value of less than 1 indicated synergism between the two compounds [61,62].

### 4.13. Statistical Analysis

Raw data were processed using Microsoft Excel (https://www.microsoft.com/en-us/microsoft-365/excel, accessed on 8 May 2024) prior to being transferred to Graphpad Prism (La Jolla, CA, USA, www.graphpad.com, accessed on 8 May 2024). Experiments depicted in Figure 1, Figure 2D, Figure 5B, Figure 9B and Figure S1A,B were analyzed by one-way ANOVA with Tukey’s multiple comparisons post-hoc test. Experiments depicted in Figure 2B, Figure 4, Figure 5D, Figure 6, Figure 7 and Figure 9D,F were analyzed by *T*-test. Experiments depicted in Figure 2A, Figure S2A,B were analyzed by linear regression and comparison of fits using Graphpad Prism. We utilized the Chou-Talalay method (isobologram and combination index) to evaluate synergy as illustrated in Figure 8B [62]. Statistical analysis was performed for experiments using Graphpad Prism. Calculated *p*-values were included to determine statistical significance (**** = *p* value < 0.0001; *** = *p* value < 0.001; ** = *p* value < 0.01; * = *p* value < 0.05; ns = not significant).

## Data Availability

All datasets generated or analyzed for this study are included in the manuscript and the Supplementary Files.

## Supplementary Materials

The following supporting information can be downloaded at: https://www.mdpi.com/article/10.3390/ijms25137003/s1.

## Abbreviations

AR^neg^-AI	Androgen-receptor-negative, androgen-independent
AR^pos^-AI	Androgen-receptor-positive, androgen-independent
AR^pos^-AD	Androgen-receptor-positive, androgen-dependent
CRPC	Castration-resistant prostate cancer
ROR1	Receptor tyrosine kinase like Orphan Receptor 1
RTK	Receptor tyrosine kinase
PGG	1,2,3,4,6 Penta-O-galloyl-β-D-glucose
AR	Androgen receptor
IC_50_	Half maximal inhibitory concentration
CI	Combination index
PI3K	Phosphoinositide 3-kinase
AKT	Serine/threonine-specific protein kinase
GSK3β	Glycogen synthase kinase 3 beta
Bad	BCL2 associated agonist of cell death
XIAP	X-linked inhibitor of apoptosis protein
MMP	Matrix metalloproteinase
