# Supplementary material for: Pentagalloyl Glucose (PGG) Exhibits Anti-Cancer Activity against Aggressive Prostate Cancer by Modulating the ROR1 Mediated AKT-GSK3β Pathway
Source: Int J Mol Sci. 2024 Jun 26;25(13):7003. doi: 10.3390/ijms25137003 (PMC11241829; doi:10.3390/ijms25137003)
Supplement: Supplementary file 1 [file ijms-25-07003-s001.zip › ijms-3027887-supplementary.pdf]

Supplementary Materials

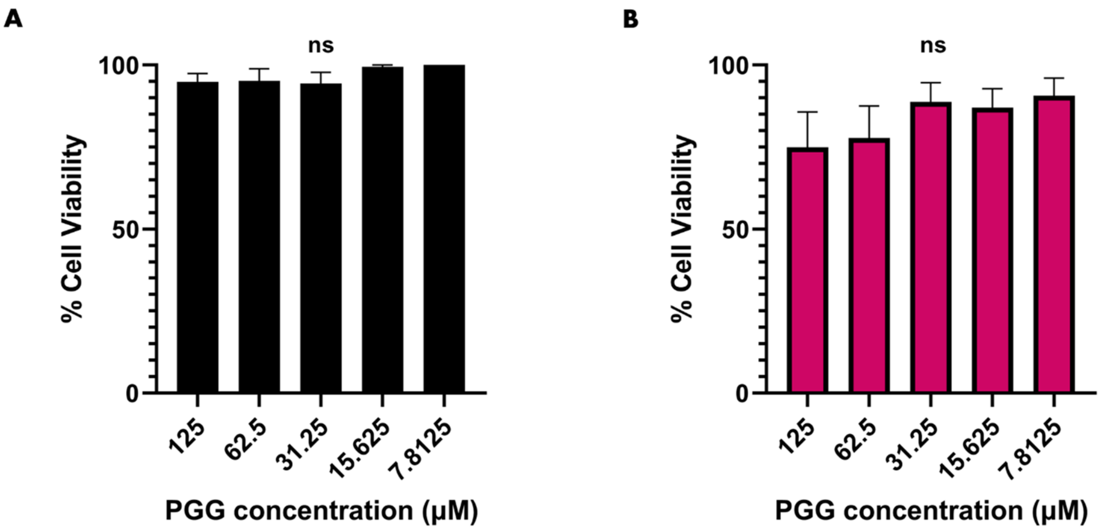

Figure S1. PGG does not reduce the viability of PC3 and RWPE-1 cells after 24 hours of treatment.
A) Cell viability of PC3 after treatment with varying concentration of PGG for 24 hours. **B)** Cell viability of RWPE-1 after treatment with varying concentrations of PGG for 24 hours. [N≥3. ns = not significant]

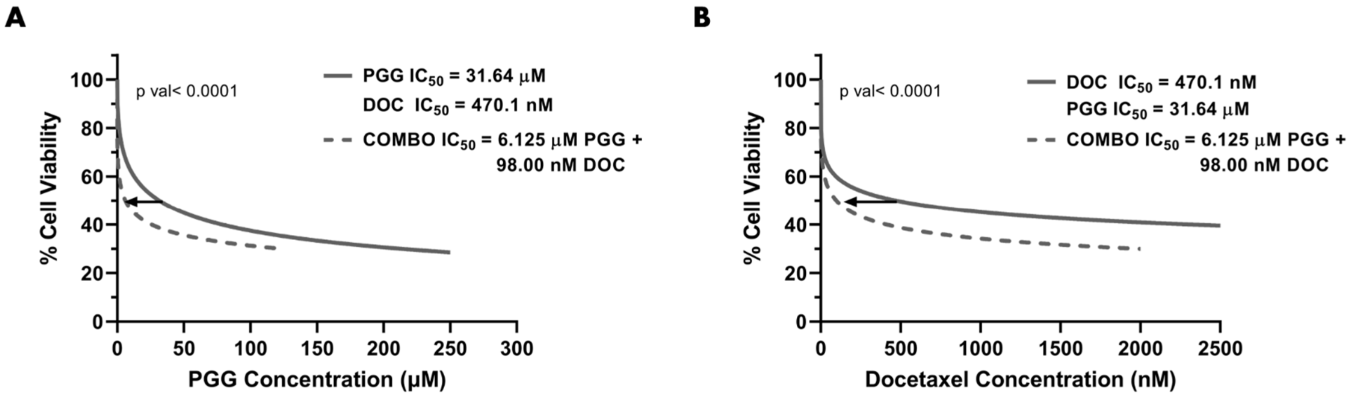

Figure S2. PGG and docetaxel combination treatment is synergistic and lowers the IC_{50} of both compounds in PC3 cells.
A) Cell viability of PC3 treated with PGG and docetaxel for 72 hours. Data are plotted against PGG concentration. **B)** Cell viability of PC3 treated with PGG and docetaxel for 72 hours. Data are plotted against docetaxel concentration. [N≥3]
